# The evolving definition of plant cell type

**DOI:** 10.3389/fpls.2023.1271070

**Published:** 2023-08-25

**Authors:** Sahand Amini, Jeffrey J. Doyle, Marc Libault

**Affiliations:** ^1^ Center for Plant Science Innovation, Department of Agronomy and Horticulture, University of Nebraska-Lincoln, Lincoln, NE, United States; ^2^ School of Integrative Plant Science, Plant Biology Section, Cornell University, Ithaca, NY, United States; ^3^ School of Integrative Plant Science, Plant Breeding & Genetics Section, Cornell University, Ithaca, NY, United States; ^4^ Single Cell Genomics Core Facility, Center for Biotechnology, University of Nebraska-Lincoln, Lincoln, NE, United States

**Keywords:** cell type, cell state, cell transition, cellular heterogeneity, single-cell technology, cell lineage, cell atlas, transcriptomics

## Introduction

**Figure 1:**
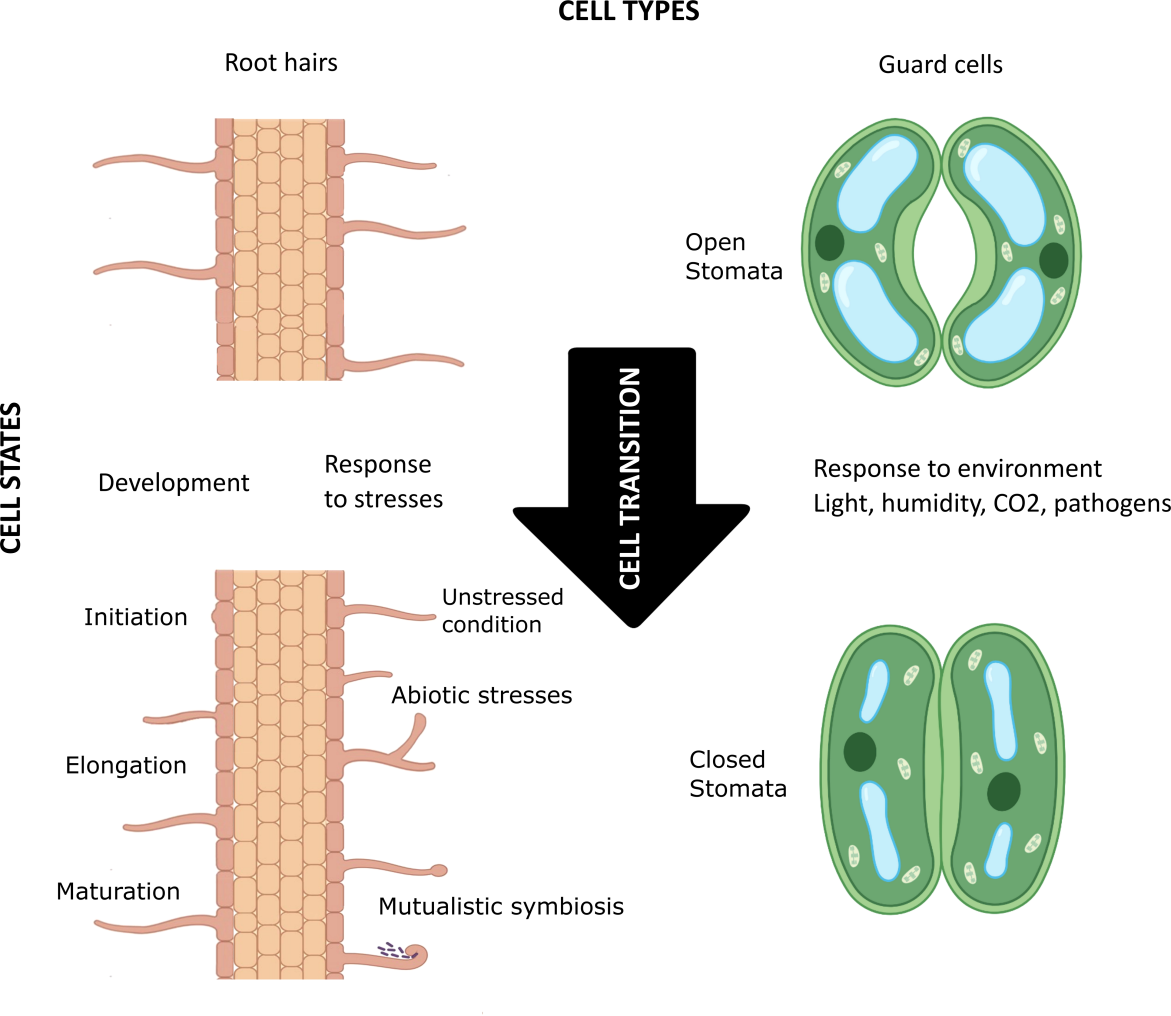
Schematic visualization of the concept of cell types, cell states, and cell transition in plants. The root hair (left) and guard cells (right) are highly differentiated plant cell types that go through various cellular transitions when developing (e.g., in initiation, elongation, and maturation of root hair) and in response to environmental factors (e.g., biotic and abiotic stresses, mutualistic symbiosis, and light/humidity/CO_2_ levels in the environment). These constant transitions lead to different cell states for each cell type. Created with BioRender.com.

In 1665 Robert Hooke, looking at cork through his microscope, discovered that plants are composed of elementary structures he named “cells”. Variation in the expression of a single genome in a complex eukaryotic organism guides the initiation, maturation, physiology, and biochemistry of cells with different shapes and sizes, playing different structural and functional roles in space and time. How many kinds of cells—”cell types”—an organism possesses of course depends on the organism’s cellular complexity, but the plasticity within a cell type fuels the emergence of the concept of cell state (**Figure 1**). The transition between cell states is driven by the developmental processes of multicellular organisms (e.g., cell determination and differentiation) and their response to environmental stresses (Wang et al., 2018). In the last decade, single cell/nucleus (sc/sn) omics, especially scRNA-seq, and spatial transcriptomics have enabled high-resolution mapping of molecular profiles of each cell, as well as mirroring cell trajectories through different states. Furthermore, unsupervised clustering of cells based on their transcriptomic (and/or epigenomic) signatures has proven effective in discovering previously unidentified cell types and cell states and providing new insight into cellular heterogeneity within a cell type (Schaum et al., 2018; Liu et al., 2021; Schumacher et al., 2021; Elmentaite et al., 2022). Classifying cells is necessary to conceptualize the biological complexity of some organs like the human brain or the plant root. But how should this be done? What criteria should be used, or, if several criteria are used, how should they be prioritized?

Although plants have provided foundational model systems for key advances in biology (e.g., Bock et al., 2023)—Mendel’s peas and Barbara McClintock’s maize transposons come immediately to mind, not even the best-developed plant models have access to anything approaching the resources poured into biomedical research on human and other animal models. Today, although rapid progress is being made in plant cell biology (Ryu et al., 2021; Xu and Jackson, 2023), human cell biology is still more advanced. A few years ago, the journal *Cell Systems* asked 15 prominent cell biologists for their “conceptual definition of ‘cell type’ in a mature organism”. Their answers varied widely; although most agreed that “cell type” was a critical concept, at least one felt that only cell states exist (Clevers et al., 2017). This diversity of opinions on how to fit natural variation into human-designed categories has been likened to the problem of defining “species” (reviewed by Doyle, 2022). How has that debate evolved, and what can plant scientists learn from it?

## Current opinions in animal single-cell biology

Given the proliferation of animal cell atlases (e.g., Packer et al., 2019; Mittnenzweig et al., 2021; Li et al., 2022; Jain et al., 2023), one might assume that the problem of cell type definition has been resolved. But Li et al. (2022) note that cell type definition remains a key challenge for atlases. It is generally agreed that the definition of a cell type should reflect intrinsic properties (i.e., the final fate and role of each cell in the body) and transient behaviors of each cell type (Morris, 2019). Cell biologists find this concept useful to have a dynamic, inclusive, and multifaceted definition of cell type, where facets include cell origin and lineage history, function, morphology, location, interactions, and molecular features (including genetic variation, epigenome, transcriptome, posttranscriptional modifications, non-coding RNAome, proteome, post-translational modifications, metabolome, and cellular localization). As with defining species, implementation is difficult when traits are incongruent, and researchers prioritize them differently (Doyle, 2022).

The prevalent approach to define cell identity is to use sc/snRNA-seq data to identify “transcriptomic cell types” (Yao et al., 2021; Zeng, 2022). For example, the *Drosophila* cell atlas defines a cell type as “a transcriptomic cluster detected at any clustering resolution that could be separated by the expression of known marker genes from other clusters” (Li et al., 2022). Such clusters can be related to one another in various ways, for example by creating a cellular equivalent of the periodic table of elements (Xia and Yanai, 2019; Moroz, 2021) or more commonly by a dendrogram (e.g., Zeng, 2022). Such a hierarchical system allows the recognition of cell types at various levels of granularity (Fishell and Kepecs, 2020). Such an approach allowed the authors of an atlas of the human intestine to group “an immense diversity of phenotypically and morphologically distinct cell types” into a small number of “broad cell types” (e.g., immune vs. stromal) whose relative frequencies varied among spatially restricted “neighborhoods” along the intestinal tract (Hickey et al., 2023). Similar approaches are found in other atlases that comprise the Human BioMolecular Atlas Program (HuBMAP; Jain et al., 2023).

It is hypothesized that what defines “cell types” are repertoires of transcription factors (TFs; Hobert et al., 2016; Lambert et al., 2018; Liang et al., 2018; Almeida et al., 2021; Isbel et al., 2022). A major goal of the field of gene regulation is to understand how cellular variation arises, regardless of whether variants are called types or states, involving an evo-devo perspective and physiological response (what cell types are shared across species, how cell types differ from one another, and how the same cell types vary among species both in their “ground state” and in their responses to stimuli). Underlying this goal is a different conception of “cell type”, one that is explicitly evolutionary, dealing with homology (similarity due to common descent). Arendt et al. (2019) define a cell type as “a set of cells accessing the same regulatory program driving differentiation” and related to the same cell type in other species by evolutionary modifications. Members of a cell type share a core regulatory complex (CoRC) comprising a group of interacting transcription factors and other proteins (Arendt et al., 2016) that is challenging to identify but can be approximated using co-expressed TFs (Almeida et al., 2021).

Recently, Domcke and Shendure (2023) proposed an explicitly lineage-based definition of cell types and their relationships that they contend will serve cell biology better than transcriptional cell types organized into atlases. In looking for an organizing principle for cell types, they reject the transcriptomic approach as inherently phenetic and thus suffering from arbitrariness in defining entities, as is true in species biology (Doyle, 2022), as well as lacking an objective means of distinguishing state from type. However, they also reject the Arendt et al. (2019) evolutionary approach as being impractical due to the divergence of cell types across highly diverse species and the difficulty of defining the CoRC. They also reject methods based on subjectively defined and incompletely known cell functions (Clevers et al., 2017; Morris, 2019). Instead, they propose to use a combination of developmentally staged whole-organism scRNA-seq approaches (e.g., Wagner et al., 2018) and newly developed lineage tracing methods (e.g., Weinreb et al., 2020) to construct lineage trees, building on a “phylodynamic” approach articulated by Stadler et al. (2021). Because development of an organism is a robust and reproducible process, such a “consensus ontogeny” of cell types would summarize the totality of an organism’s development, with each cell tracing back to the zygote. Additional molecular features (e.g., sc transcriptome/epigenome or expression of key TFs) could be mapped onto this tree to group cells into types by showing variation within and among individuals at molecular and phenotypic states. The aspirational goal for complex animal systems is the resolution currently attainable in *C. elegans*, with its fully resolved fate map (Sulston et al., 1983; Packer et al., 2019).

We conclude that although the transcriptomic cell type concept (Zeng, 2022) is prevalent in the animal cell atlas community, the approach has recognized limitations. Thus, the debate over what constitutes a cell type in theory, and how to identify cell types in practice, is likely to continue for the foreseeable future, like the debate over species (Doyle, 2022).

## Discussion

Recent reviews of plant single-cell biology (e.g., Ryu et al., 2021; Bawa et al., 2022; CuPerus, 2022; Xu and Jackson, 2023) use the term “cell type” extensively, but do not define the term explicitly, and in this they follow the papers they cite. For example, Xu and Jackson (2023) list 35 flowering plant single-cell studies, most on *Arabidopsis* and maize, none of which include a precise definition of “cell type”. Implicitly, papers using sc/snRNA-seq methods employ the transcriptomic cell type (Zeng, 2022), with novel cell types being defined by the absence of expression of well-characterized marker genes. Accordingly, although there is interest in the regulatory underpinnings of cell types (e.g., Dorrity et al., 2021; Nobori et al., 2023), the dominant trend of these 35 papers is to consider cell type definition synonymous with successful assignment of cell clusters produced by dimensionality reduction (e.g., UMAP) to anatomically/microscopically known cell types. The lack of cell-type-specific marker genes is thus seen as a key limitation of current plant cell studies (CuPerus, 2022).

But just as is true with animals, this ignores the distinction between cell type vs. cell state. A plant cell cluster need not be synonymous with a cell type, and as with the human cell atlases (e.g., Hickey et al., 2023) plant researchers can choose a preferred degree of granularity. For example, Coate et al. (2020) studied paralogous gene subfunctionalization using the *Arabidopsis* root single-cell data of Ryu et al. (2019), who had recognized 19 different cell clusters, but subdivided these further into 36 clusters. Given the concerns about the ability of dimensionality reduction methods to produce meaningful groupings (Chari et al., 2021), objective criteria that could guide the interpretation of the relationship of cell clusters to cell types or cell states would be welcome. It is worth noting that Marand et al. (2021) used machine learning to identify combinations of TFs likely to underlie cell type specification, approximating a CoRC approach (Arendt et al., 2019).

Human cell atlases now routinely incorporate spatial transcriptomics approaches (Jain et al., 2023), and this level of resolution is now possible in plants, as well (Giacomello, 2021; Nobori et al., 2023; Yu et al., 2023). For example, recently Cervantes-Pérez et al. (2023) and Sun et al. (2023) produced high-resolution spatial transcriptomic atlases of soybean symbiotic root nodules. But is an atlas approach based on the transcriptomic cell type definition the best way forward, or should lineage and tree-based alternatives be considered? Model plants tractable for the whole-organism molecular analyses at single-cell level recommended by Domcke and Shendure (2023) include the tiny duckweed (*Spirodela*; Wu et al., 2023) and, beyond flowering plants, the liverwort *Marchantia* (Bowman et al., 2022). Plants have some advantages over animals for lineage tracing, notably that with few exceptions plant cells are immobile. Donà et al. (2023) recently reported proof of concept CRISPR-based lineage tracing in *Arabidopsis* roots, and further showed its utility in *Marchantia*, concluding that plant lineage tracing can generate “a comprehensive visual map of differentiating cell files and tissue fate and help to characterize the role of key genes at developmental branch points.”

In conclusion, the transcriptomic definition of a cell type is currently dominant in the era of single cell multiomics, both in animals and in plants. It is currently a useful concept for many practical purposes. But “scientific progress accelerates when paradigm shifts occur”, and we assume that the definition of cell type will evolve, ultimately illustrating this maxim for plant biology.

## Author contributions

SA: Conceptualization, Writing – original draft, Writing – review & editing. JD: Conceptualization, Writing – review & editing. ML: Conceptualization, Funding acquisition, Writing – review & editing.
